# Targeting G_i/o_ protein–coupled receptor signaling blocks HER2-induced breast cancer development and enhances HER2-targeted therapy

**DOI:** 10.1172/jci.insight.150532

**Published:** 2021-09-22

**Authors:** Cancan Lyu, Yuanchao Ye, Maddison M. Lensing, Kay-Uwe Wagner, Ronald J. Weigel, Songhai Chen

**Affiliations:** 1 Departments of Neuroscience and Pharmacology, Roy J. and Lucille A. Carver College of Medicine, University of Iowa, Iowa City, Iowa, USA.; 2 Department of Oncology, Wayne State University School of Medicine, Detroit, Michigan, USA.; 3 Department of Surgery, Roy J. and Lucille A. Carver College of Medicine, University of Iowa, Iowa City, Iowa, USA.

**Keywords:** Oncology, Therapeutics, Breast cancer, G protein&ndash;coupled receptors, Signal transduction

## Abstract

GPCRs are highly desirable drug targets for human disease. Although GPCR dysfunction drives development and progression of many tumors, including breast cancer (BC), targeting individual GPCRs has limited efficacy as a cancer therapy because numerous GPCRs are activated. Here, we sought a new way of blocking GPCR activation in HER2^+^ BC by targeting a subgroup of GPCRs that couple to G_i/o_ proteins (G_i/o_-GPCRs). In mammary epithelial cells of transgenic mouse models, and BC cell lines, HER2 hyperactivation altered GPCR expression, particularly, G_i/o_-GPCR expression. G_i/o_-GPCR stimulation transactivated EGFR and HER2 and activated the PI3K/AKT and Src pathways. If we uncoupled G_i/o_-GPCRs from their cognate G_i/o_ proteins by pertussis toxin (PTx), then BC cell proliferation and migration was inhibited in vitro and HER2-driven tumor formation and metastasis were suppressed in vivo. Moreover, targeting G_i/o_-GPCR signaling via PTx, PI3K, or Src inhibitors enhanced HER2-targeted therapy. These results indicate that, in BC cells, HER2 hyperactivation drives aberrant G_i/o_-GPCR signaling and G_i/o_-GPCR signals converge on the PI3K/AKT and Src signaling pathways to promote cancer progression and resistance to HER2-targeted therapy. Our findings point to a way to pharmacologically deactivate GPCR signaling to block tumor growth and enhance therapeutic efficacy.

## Graphical abstract

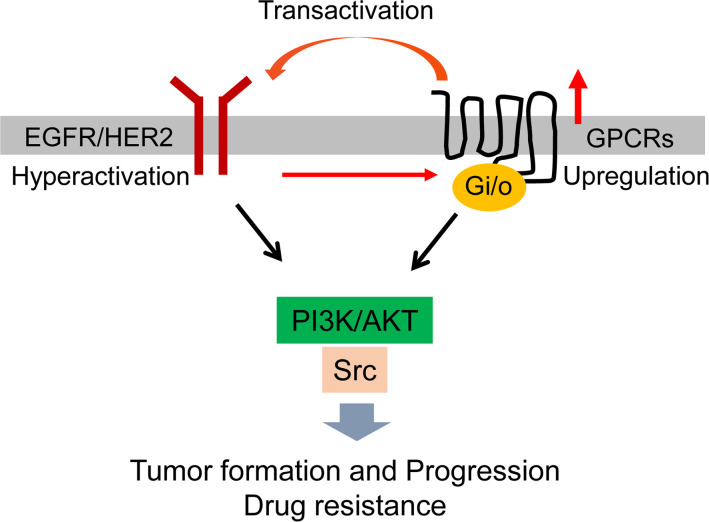

## Introduction

Breast cancer is the most common cancer and the second-most common cause of cancer death in US women. Approximately 15%–20% of all breast cancers overexpress ErbB2/HER2 and so are classified as HER2^+^ subtypes, which are associated with aggressive cancers with poor clinical outcomes (1). HER2 is a member of the ErbB family, which includes EGFR/ErbB1, ErbB2/HER2, ErbB3, and ErbB4 — all transmembrane receptor tyrosine kinases (refs. 2, 3). ErbB2/HER2 has no known ligands but can homodimerize or heterodimerize with EGFR or HER3 (4). Dimerized HER2 activates a complex cascade of downstream signaling that primarily consists of the PI3K/AKT and the MAPK pathways (4). HER2 hyperactivation induces breast tumor formation, progression, and metastasis.

The most successful treatment for HER2^+^ breast cancer is HER2-targeted therapy (5). Several FDA-approved anti-HER2 drugs, including the humanized monoclonal antibody, trastuzumab, and the small-molecule dual inhibitor of HER2 and EGFR, lapatinib, significantly improved clinical outcomes of patients with HER2^+^ breast cancer. Nevertheless, tumors that initially respond to HER2-targeted therapy can eventually develop resistance (5). To improve clinical outcomes of advanced HER2^+^ breast cancer, it is critical to develop novel therapeutic approaches that improve the efficacy of HER2-targeted therapy.

GPCRs are the largest family of cell surface receptors; they consist of over 800 members that regulate a plethora of biological functions (6). GPCR dysfunction drives the development and progression of many tumors, including breast cancer (7). Transcriptomic profiling shows breast cancer cells aberrantly express multiple GPCRs (8). In a variety of breast cancer molecular subtypes, proteogenomic analysis identifies aberrant GPCR activation (9).

In preclinical mouse models, breast tumor growth and/or metastasis is driven by diverse GPCRs (e.g., lysophosphatidic acid [LPA], thrombin, endothelin and prostaglandin E2 receptors, and many chemokine receptors, such as CXCR4 and CCR5; ref. 10). Nevertheless, attempts to target individual GPCRs as a cancer therapy have failed in many clinical trials, largely due to the lack of efficacy (11). Indeed, although GPCRs are the most desirable drug targets and nearly 40% of currently marketed drugs target GPCRs, only a few are effective for cancer (11). This failure is likely because many different GPCRs are dysregulated in cancer and all have the same oncogenic effect. Thus, to harness the therapeutic power of controlling GPCRs, we must develop approaches that overcome their redundant function. One approach is to target their shared functions in driving cancer.

Most GPCRs mediate cellular responses by activating heterotrimeric G proteins, which consist of Gα and Gβγ subunits (12, 13). Sequence homology among Gα subunits distinguishes 4 classes of G proteins: G_i/o_, G_s_, G_q/11_, and G_12/13_; although GPCRs can activate more than one class, they prefer one class over another (12). Of the 376 nonsensory human GPCRs, approximately 111 are coupled to G_i/o_, 55 are coupled to G_s_, 81 are coupled to G_q/11_, 12 are coupled to G_12/13_, and 153 have unknown G protein linkage.

In breast cancer, the G_i/o_-GPCRs appear to be particularly important. Pertussis toxin (PTx), which catalyzes the ADP ribosylation of the Gα_i/o_ subunits and selectively uncouples G_i/o_ proteins from their receptors (e.g., PAR1, LPA, and chemokine receptors; ref. 14) blocks the effects of most GPCRs implicated in cancer, particularly progression and invasion (15–19). Moreover, 2% of all breast cancers carry a constitutively active form of G_αo_ (R243H) that promotes oncogenic transformation of normal mammary epithelial cells (20, 21). Additionally, we and others previously showed that Gβγ is the point of convergence for many G_i/o_-coupled GPCRs signals mediating breast tumor cell growth and migration in vitro and tumor growth and metastasis in vivo (17, 22, 23). Thus, targeting G_i/o_-GPCRs may be an effective strategy for halting breast tumor progression and overcoming drug resistance.

In this study, we sought to determine the function of a whole set of G_i/o_-GPCRs in HER2-induced breast cancer development and assess whether targeting G_i/o_-GPCR signaling could augment HER2-targeted therapy. We show that aberrant G_i/o_-GPCR signaling promotes breast cancer cell proliferation and migration in vitro and contributes to HER2-induced tumor development and metastasis in a genetically modified mouse model. Blocking G_i/o_-GPCR signaling also enhances the efficacy of the HER2-targeted therapy in vitro and in vivo. These findings demonstrate that targeting G_i/o_-GPCR signaling may represent a new approach to blocking tumor progression and augmenting HER2-targeted therapy.

## Results

### HER2 regulates GPCR expression in mammary epithelia.

To identify GPCRs that drive breast cancer progression, we profiled invasive breast cancers from TCGA database for RNA expression of 376 nonsensory GPCRs. In multiple molecular subtypes of breast cancer, including luminal A, luminal B, HER2, and basal subtypes, each tumor sample overexpressed several GPCRs that couple to G_i/o_, G_s_, G_q/11_, G_12/13_, or unknown G proteins (Supplemental Figure 1, A–E; supplemental material available online with this article; https://doi.org/10.1172/jci.insight.150532DS1), but no single GPCR was overexpressed in all samples. However, because tumors contain mixed populations of cells, these analyses could not reveal the identity of cells that overexpress GPCRs.

**Figure 1:**
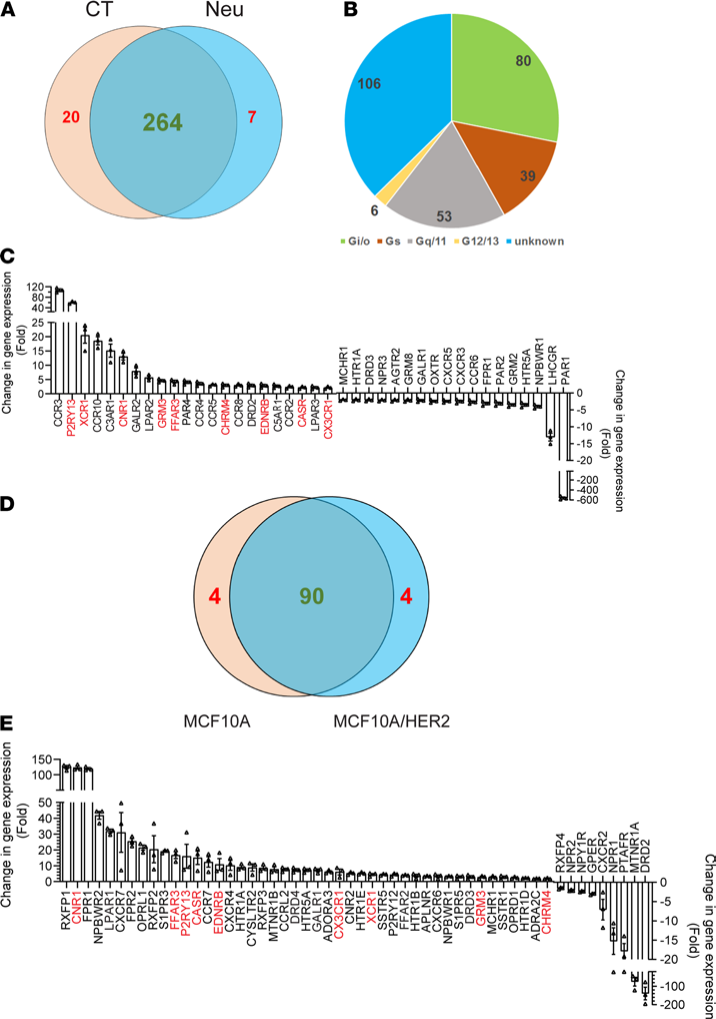
HER2 regulates GPCR expression in mammary epithelial cells. (**A**) Venn diagram showing the number of GPCRs expressed in control and Neu cells. (**B**) G protein linkage of GPCRs expressed in common in control and Neu cells. (**C**) The G_i/o_-GPCRs showing more than a 2-fold change of expression in Neu cells as compared with control cells. Data are expressed as mean ± SEM, *n =* 3. (**D**) Venn diagram showing the number of G_i/o_-GPCRs expressed in MCF10A and MCF10A/HER2 cells. (**E**) The G_i/o_-GPCRs showing more than a 2-fold change of expression in MCF10A/HER2 cells as compared with control cells. Data are expressed as mean ± SEM, *n =* 3. The common GPCRs upregulated in Neu and MCF10A/HER2 cells are highlighted in red.

Therefore, GPCR expression profiling was further done in tumors of a well-established transgenic animal model of human HER2^+^ breast cancer, Neu mice, which express an activated rat ErbB2/HER2 homologue selectively in the mammary gland (24). Mammary epithelia from the mammary glands of wild-type versus premalignant mammary tissue of age-matched Neu mice were profiled for expression of 370 nonsensory GPCRs. The results showed that 291 GPCRs were expressed by control and Neu epithelia, 264 were expressed by both, 20 were expressed in controls only, and 7 GPCRs were uniquely expressed in Neu cells (Figure 1A). Of the 264 GPCRs expressed in both normal and cancer cells, most (106 GPCRs) have unknown G protein linkages (Figure 1B). Of the rest, more couple to G_i/o_ (80 GPCRs) than to other G proteins (Figure 1B). Compared with the control, 133 GPCRs were altered more than 2-fold in Neu epithelial cells (Table 1). Among these, 44 have unknown G protein linkages, 40 couple to G_i/o_, 28 couple to G_q_, 17 couple to G_s_, and 4 couple to G_12/13_ (Figure 1C, Supplemental Figure 2, and Table 1). Of the G_i/o_-GPCRs differentially expressed in Neu mice, more were upregulated (22 G_i/o_-GPCRs) than downregulated (ref. 18, Figure 1C, and Table 1).

To test further if the altered GPCR expression is induced by HER2 overexpression and hyperactivation, MCF10A- and MCF10A-overexpressing HER2 (MCF10A/HER2) cells were profiled for expression of 106 G_i/o_-GPCRs. Both lines expressed 90 receptors and each line expressed 4 unique genes (Figure 1D). Compared with MCF10A cells, MCF10A/HER2 cells upregulated more receptors (45 upregulated more than 2-fold) than were downregulated (9 downregulated; Figure 1E). Among the 45 GPCRs upregulated in MCF10A/HER2 cells, 9 (i.e., CNR1, FFAR3, P2RY13, CASA, EDNRB, CX3CR1, XCR1, GRM3, and CHRM4) were also upregulated in Neu cells (Figure 1C), suggesting HER2 overexpression and hyperactivation alters GPCR expression in a cell type-specific manner.

To validate the function of the upregulated G_i/o_-GPCRs in MCF10A/HER2 cells, we stimulated MCF10A and MCF10A/HER2 cells with LPA and SDF1α, because the mRNA levels of their cognate receptors, LPAR1, CXCR4, and CXCR7, were upregulated at approximately 32-, 10-, and 31-fold, respectively, in MCF10A/HER2 cells versus MCF10A cells (Figure 1E). Increased protein expression of LPAR1, CXCR4, and CXCR7 in MCF10A/HER2 cells was confirmed by Western blotting (Supplemental Figure 3A). The phosphorylation of AKT^S473^ and Src^Y416^ stimulated by LPA and SDF1α increased substantially in MCF10A/HER2 cells versus MCF10A cells, while increased ERK phosphorylation was only observed by LPA stimulation (Supplemental Figure 3, B and C). Moreover, PTx treatment suppressed the AKT, Src, and ERK phosphorylation that was stimulated by LPA and SDF1α but not EGF (Supplemental Figure 3, D–F), suggesting G_i/o_ proteins, in particular, drive LPA- and SDF1α-stimulated signaling.

### G_i/o_-coupled receptor signaling promotes HER2-induced tumor growth and metastasis.

**Figure 2:**
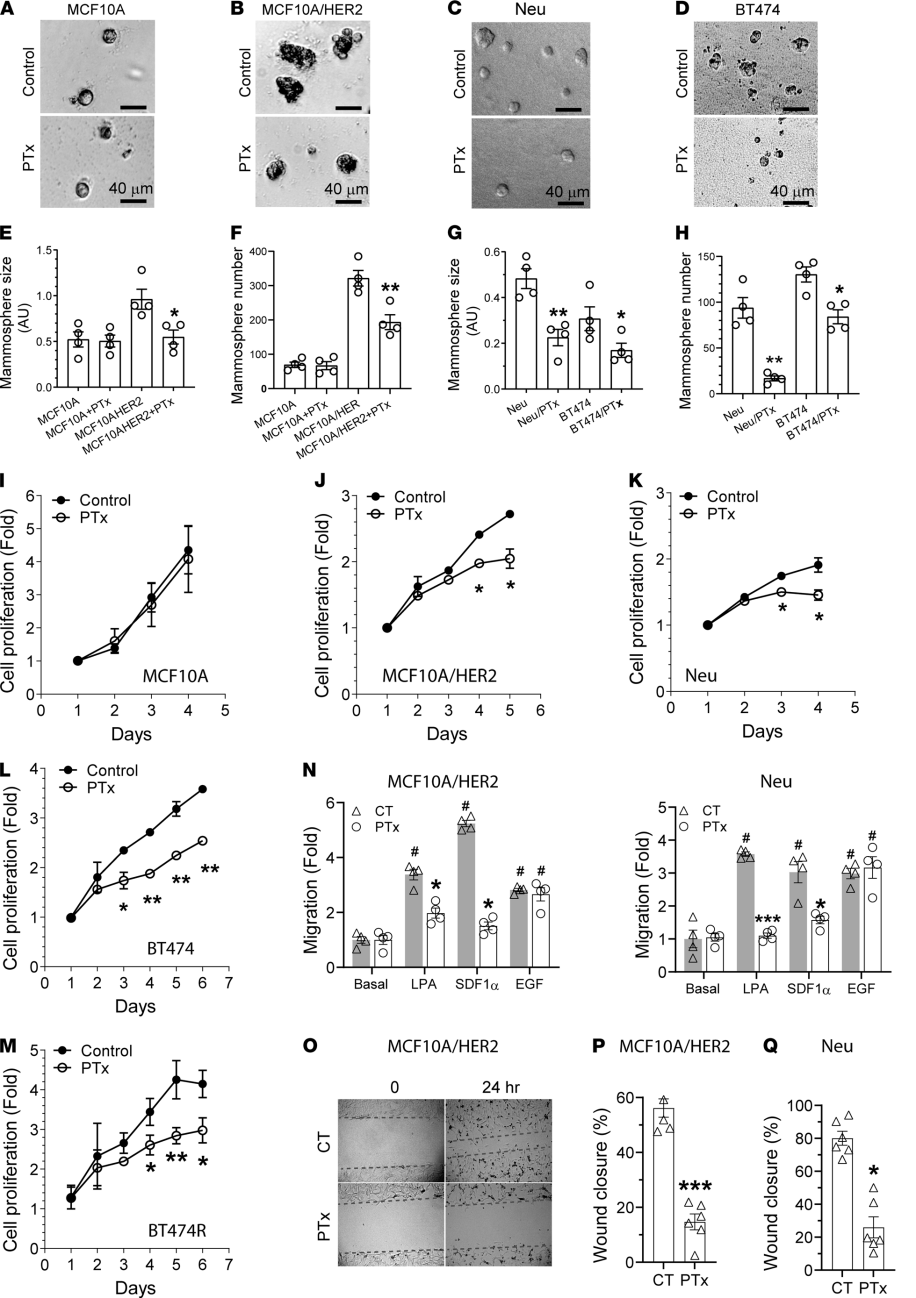
G_i/o_-GPCR signaling regulates the growth and migration of HER2^+^ mammary epithelial cells. (**A**–**D**) Representative images showing MCF10A (**A**), MCF10A/HER2 (**B**), Neu (**C**), and BT474 (**D**) cells grown in Matrigel treated with vehicle control (CT) or PTx (0.2 μg/ml). Scale bar: 40 μm. (**E**–**H**) Quantitative data showing the size (**E** and **G**) and number (**F** and **H**) of MCF10A, MCF10A/HER2, Neu, and BT474 mammospheres. *P < 0.05, **P < 0.01, ***P < 0.001, vs. MCF10A/HER2, Neu, or BT474, *n =* 4. (**I**–**N**) The effect of PTx treatment on proliferation (**I**–**M**) and LPA-, SDF1α-, and EGF-induced Transwell migration (**N**) of the indicated cells. *P < 0.05, **P < 0.01, ***P < 0.001 vs. control; ^#^*P <* 0.05 vs. basal; *n =* 4–6. (**O**) Representative images showing the size of the wound at 0 and 24 hours in MCF10A/HER2 cells treated with vehicle control or PTx. Original magnification, ×10. (**P** and **Q**) Quantitative data showing the effect of PTx on wound healing in MCF10A/HER2 (**P**) and Neu (**Q**) cells. *P < 0.05, ***P < 0.001 vs. control, *n =* 4–6. Two-tailed unpaired Student’s *t* test was used for all statistical analysis in this figure, except for **N**, which was analyzed by two-way ANOVA.

To test whether G_i/o_-GPCRs drive HER2^+^ breast cancer proliferation, we determined the effect of PTx treatment on MCF10A and MCF10A/HER2 cell growth in Matrigel. Here, MCF10A cells formed small, round, and well-organized acini, whereas MCF10A/HER2 cells formed large and disorganized colonies with multiple protrusions (Figure 2, A and B). PTx treatment neither changed the size nor the number of MCF10A acini, but it significantly reduced the size and number of MCF10A/HER2 colonies (Figure 2, E and F). Similarly, treatment of Neu and BT474 cells also decreased the size and number of mammospheres that could grow in Matrigel (Figure 2, C, D, G, and H). PTx treatment did not affect MCF10A proliferation in 2D culture but inhibited proliferation of several HER2^+^ breast cancer cell lines, including Neu, MCF10A/HER2, BT474, and BT474R, a trastuzumab-resistant BT474 derivative (Figure 2, I–M). These findings suggest that G_i/o_-GPCR signaling contributes to HER2-induced mammary tumor cell growth but is dispensable for normal mammary epithelial cell growth.

PTx treatment also decreased Transwell migration of MCF10A/HER and Neu cells induced by LPA and SDF1α (Figure 2N). EGF-induced cell migration was not affected by PTx, which was expected because EGF activates EGFR in a G protein–independent manner (Figure 2N and Supplemental Figure 3F). In the wound-healing assay, MCF10A/HER and Neu cell migration was also inhibited (Figure 2, O–Q), indicating that G_i/o_-GPCR signaling drives mammary tumor cell migration.

**Figure 3:**
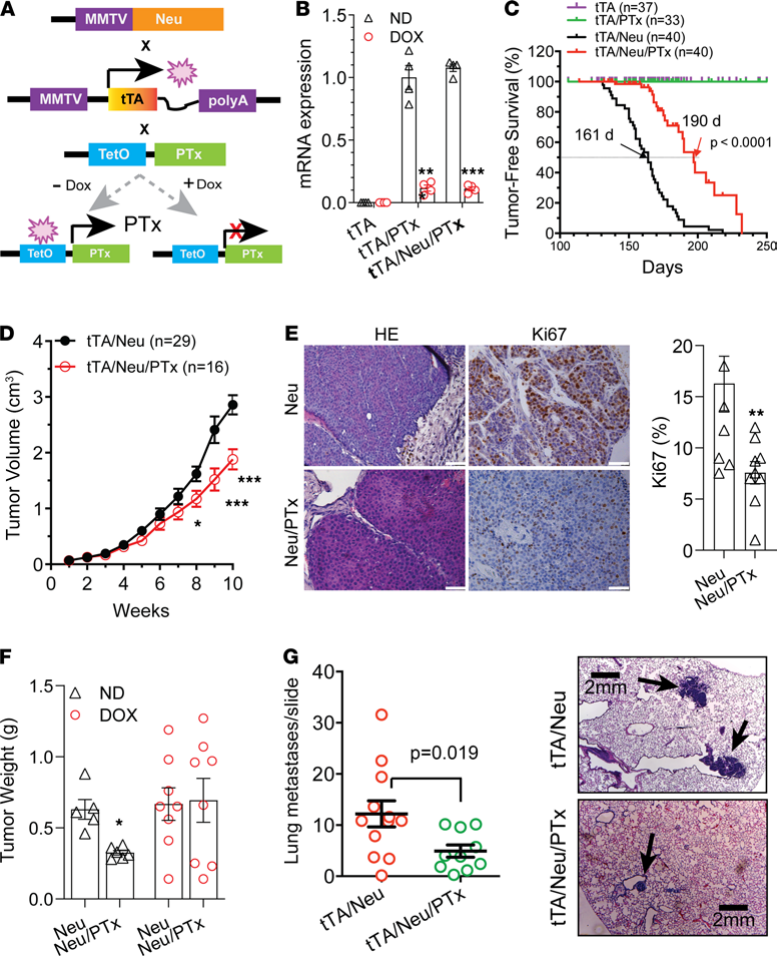
G_i/o_-GPCR signaling contributes to HER2-induced mammary tumor development. (**A**) Schematic representation of mouse breeding and the regulation of PTx expression via doxycycline (Dox). (**B**) qPCR results showing inducible PTx expression by doxycycline in mammary epithelial cells. **P < 0.01, ***P < 0.001 vs. Dox, *n =* 4. (**C**) Tumor-free survival curves. (**D**) Neu and Neu/PTx tumor growth curves. The size of the largest tumor was monitored once weekly by caliper. *P < 0.05, ***P < 0.001 vs. tTA/Neu. (**E**) Representative images showing H&E and Ki67 staining of Neu and Neu/PTx tumors. Quantitative data for Ki67 staining are shown. Scale bar: 30 μm. **P < 0.01 vs. Neu, *n =* 9. (**F**) The weight of tumors grown from Neu and Neu/PTx tumor cells in mice fed with normal chow (ND) or doxycycline-containing chow. *P < 0.05 vs. Neu, *n =* 5–8. (**G**) The number of lung metastases and images representative of H&E-stained lung metastases (indicated by arrows) from transgenic Neu and Neu/PTx mice. Scale bar: 2 mm. Two-tailed unpaired Student’s *t* test was used for all statistical analysis in this figure.

To corroborate these findings in vivo, we crossed TetO-PTx–transgenic mice (which carry a catalytic subunit of PTx under a tetO promoter) with MMTV-tTA–transgenic mice (which express the transactivator tTA from the mammary gland-specific MMTV promoter; Figure 3A). Their bigenic offspring (tTA/PTx; TPTx) were crossed to Neu mice to generate trigenics, tTA/Neu/PTx (Neu/PTx) mice. qPCR analysis confirmed that PTx expression in their mammary glands and tumors was under doxycycline control (Figure 3B and data not shown). Moreover, the PTx expression neither affected the litter size nor the survival of pups (data not shown); whole-mount in situ staining of mammary glands did not detect a difference between mice with various genotypes in the number of terminal end buds at 1 month or the length of ductal distance in the mammary glands at different ages (Supplemental Figure 4, A–C). These data confirm that PTx did not affect mammary gland development. PTx expression, however, significantly delayed Neu-induced mammary tumor formation (161 days vs. 190 days) and reduced tumor growth (Figure 3, C and D). Ki67 staining showed that the percentage of Ki67^+^ tumor cells was significantly lower in Neu/PTx tumors than Neu tumors (16.14 ± 2.99 vs. 8.41 ± 2.0, *P <* 0.05), suggesting tumor cell proliferation was inhibited by PTx (Figure 3E). To validate the inhibition of Neu tumor growth by PTx, we isolated Neu and Neu/PTx tumor cells and implanted an equal number of them into FVB/N mice fed normal or doxycycline-containing chow (to control PTx expression). In mice fed normal chow, Neu cells grew larger tumors than Neu/PTx cells (Figure 3F). This difference in tumor growth was abolished in mice fed doxycycline-containing chow (which suppressed PTx expression; Figure 3F).

Lung metastases were monitored when primary tumors reached a similar size (a diameter of ~2.0 cm), showing that coexpression of PTx with Neu also reduced the size and number of lung metastases (Figure 3G). These findings indicate that G_i/o_-GPCR signaling contributes to HER2-induced tumor initiation, progression, and metastasis.

### G_i/o_-GPCRs crosstalk with EGFR and HER2.

**Figure 4:**
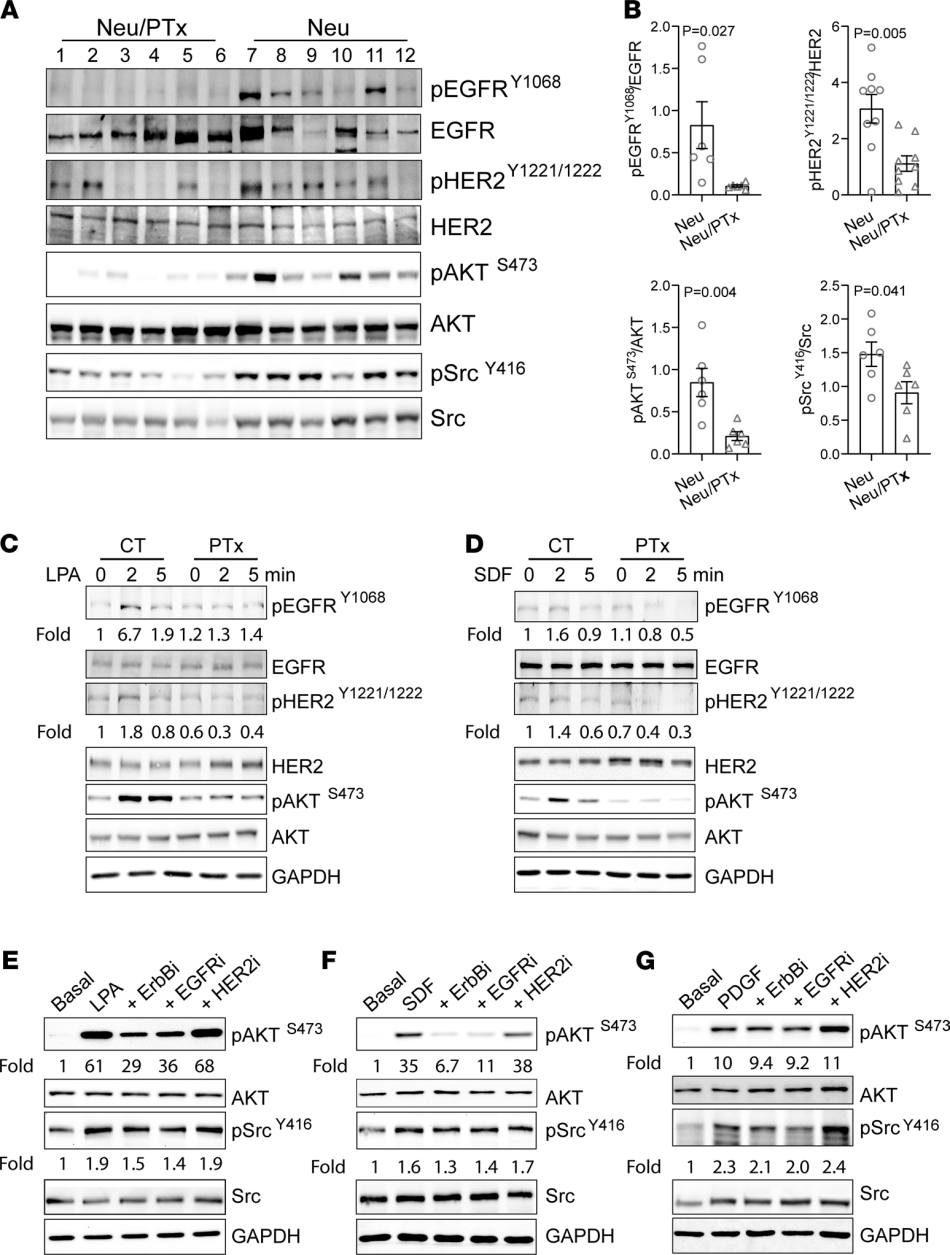
G_i/o_-GPCRs induce transactivation of EGFR and HER2 in mammary tumor cells. (**A** and **B**) Western blotting (**A**) showing decreased phosphorylation of EGFR^Y1806^, HER2^Y1221/1222^, AKT^s473^, and Src^Y416^ in Neu/PTx tumors as compared with Neu tumors. Each lane represents one sample from individual tumors. (**B**) The Western blot data from **A** were quantified and expressed as the ratio of the phosphorylated to total proteins. Two-tailed unpaired Student’s *t* test was used for statistical analysis of the data in **B**, and *P* values are shown. (**C** and **D**) Western blotting showing phosphorylation of EGFR, HER2, and AKT in Neu cells treated with vehicle control or PTx and stimulated with LPA (**C**) or SDF1α (**D**). (**E**–**G**) The effect of 1 μM of a pan-ErbB– (sapitinib), EGFR- (erlotinib), or HER2- (cp-724714)specific inhibitor on LPA- (**E**), SDF1α- (**F**) or PDGF-stimulated (**G**) AKT and Src phosphorylation in Neu cells. The phosphorylation of EGFR^Y1068^, HER2^Y1221/1222^, AKT^S473^, and Src^Y416^ was quantified as the ratio of the phosphorylated to total proteins and expressed as the fold increase over basal, which is indicated underneath the images. The images are representatives of at least 3 independent experiments and were assembled from multiple blots run with the samples from the same experiments.

To identify mechanisms by which G_i/o_-GPCRs regulate HER2 tumor development and progression, we assessed the activation status of EGFR and HER2 and their downstream effectors. Immunoblotting found that, compared with Neu tumors, Neu/PTx tumors showed significantly reduced phosphorylation of EGFR and HER2, AKT^S473^, and Src^Y416^ (Figure 4, A and B). The results were validated by IHC analysis (Supplemental Figure 5A), suggesting that G_i/o_-GPCRs likely regulate EGFR and HER2 activation in tumor cells.

To test if G_i/o_-GPCRs regulate EGFR and HER2 activities via transactivation, Neu cells were stimulated with LPA or SDF1α and then probed for EGFR and HER2 phosphorylation (because these cells expressed the cognate receptors for LPA, SDF1α, LPAR1, and CXCR4; Supplemental Figure 3A). As shown in Figure 4, C and D, LPA and SDF1α stimulated EGFR, HER2, and AKT phosphorylation, which was abolished by PTx, indicating the involvement of G_i/o_ proteins. Similar results were found in MCF10A/HER2 cells (Supplemental Figure 5, B and C) and BT474 cells, which endogenously express HER2 (Supplemental Figure 5, D and E).

Next, we assessed whether EGFR and HER2 transactivation by G_i/o_-GPCRs drives activation of effectors shared by G_i/o_-GPCRs and EGFR/HER2, such as AKT and Src. Neu and MCF10A/HER2 cells were treated with a pan-ErbB–, EGFR-, or HER2-specific inhibitor, sapitinib (25), erlotinib (26), or cp-724714 (27), respectively; the specificity of these inhibitors was confirmed in Neu cells stimulated with EGF. As expected, EGF-stimulated phosphorylation of EGFR and HER2 was largely abolished by the pan-ErbB– and EGFR-specific inhibitors, Sapitinib and erlotinib (Supplemental Figure 5F). In contrast, the HER2-specific inhibitor, cp-724714, only inhibited EGF-stimulated HER2 phosphorylation, consistent with the idea that HER2 heterodimerizes with activated EGFR to form a signaling complex (Supplemental Figure 5F). As shown in Figure 4, E and F, and Supplemental Figure 5, G and H, LPA- and SDF1α-stimulated AKT phosphorylation was partially suppressed by sapitinib and erlotinib in both Neu and MCF10A/HER2 cells, while Src phosphorylation was not significantly affected in either cells. The HER2-specific inhibitor did not affect LPA- and SDF1α-stimulated AKT and Src phosphorylation in either cell line. The effects of these inhibitors on LPA- and SDF1α-stimulated signaling are likely specific, because they had little effect on PDGF-stimulated AKT and Src phosphorylation in Neu cells (Figure 4G). These findings suggest that transactivation of EGFR by G_i/o_-GPCRs contributes to the activation of selective effectors in a GPCR- and pathway-dependent manner.

### Targeting G_i/o_-GPCR signaling by PTx enhances HER2-targeted therapy.

**Figure 5:**
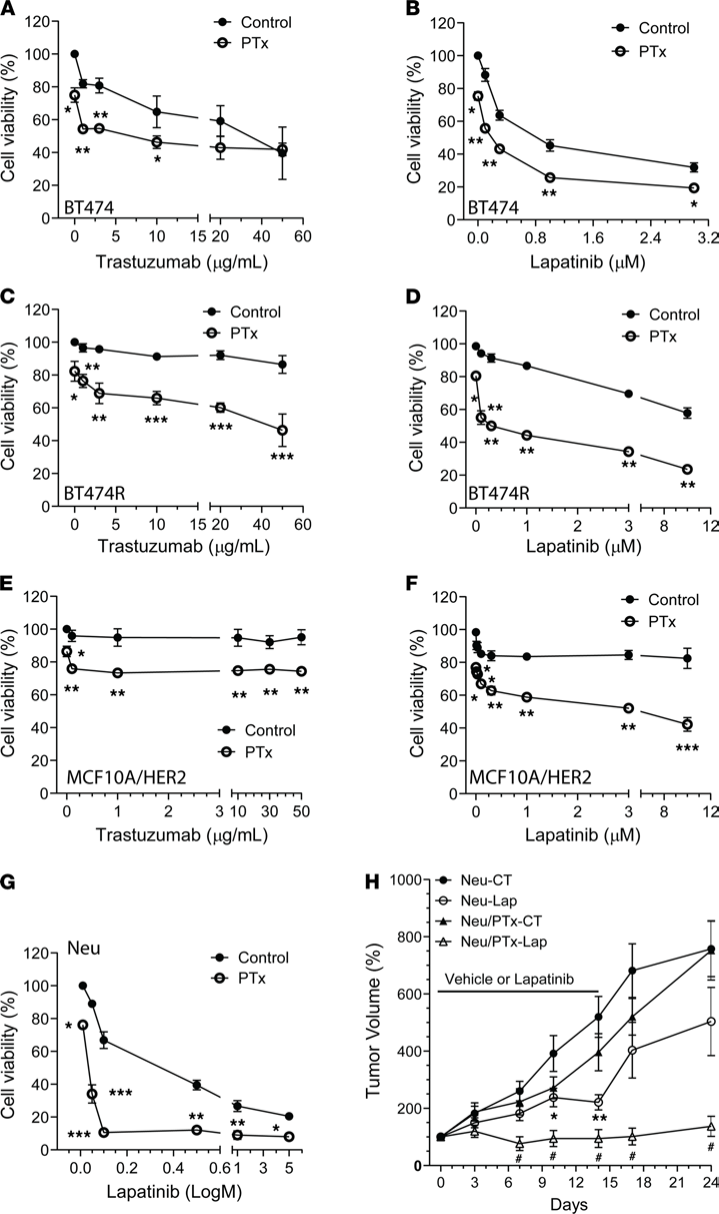
Targeting G_i/o_-GPCR signaling by PTx enhances HER2-targeted therapy. (**A**–**G**) The effect of PTx (0.2 μg/ml) on the viability of BT474 (**A** and **B**), BT474R (**C** and **D**), MCF10A/HER2 (**E** and **F**), and Neu (**G**) cells treated with varying concentrations of trastuzumab for 5 days or lapatinib for 3 days. *P < 0.05, **P < 0.01, ***P < 0.001 vs. control (CT), respectively, *n =* 3–9. (**H**) The growth curves of Neu and Neu/PTx tumors treated with vehicle control or lapatinib (Lap; 200 mg/kg, daily gavage). *P < 0.05, **P < 0.01 vs. Neu-CT; ^#^*P <* 0.05 vs. Neu-Lap, *n =* 6–9. Two-tailed unpaired Student’s *t* test and one-way ANOVA were used for statistical analysis of data in **A**–**G** and **H**, respectively.

Trastuzumab and lapatinib are the major anti-HER2 therapeutic reagents for HER2^+^ breast cancer (5). Because blocking G_i/o_-GPCR signaling suppressed HER2-induced tumor growth, we tested if targeting G_i/o_-GPCRs affects the therapeutic efficacy of trastuzumab and lapatinib in Neu, MCF10A/HER2, BT474, and BT474R cells. After 5 days of treatment, trastuzumab significantly inhibited BT474 cell proliferation in a dose-dependent manner but had little effect on the growth of the other cells tested (Figure 5, A, C, and E). Both BT474 and Neu cells were sensitive but BT474R and MCF10A/HER2 cells were relatively resistant to lapatinib (Figure 5, B, D, F, and G). Notably, cotreatment with PTx enhanced the potency and/or efficacy of trastuzumab and lapatinib in all cells tested (Figure 5 and Table 2).

To corroborate our findings in vivo, FVB mice were implanted with Neu and Neu/PTx tumors and maintained until the tumors grew to a comparable size (~100 mm^3^); for 2 weeks afterward, mice were treated by daily oral gavage with vehicle control or lapatinib (200 mg/kg). Compared with the control, lapatinib partially suppressed the growth of Neu tumors but completely blocked growth and even caused regression of Neu/PTx tumors (Figure 5H). Once the lapatinib treatment was stopped, Neu tumors resumed growth at an accelerated rate, whereas growth of the Neu/PTx tumors remained largely suppressed, over 10 days (Figure 5H).

### Combination with PI3K and Src inhibitors enhances the HER2-targeted therapy.

**Figure 6:**
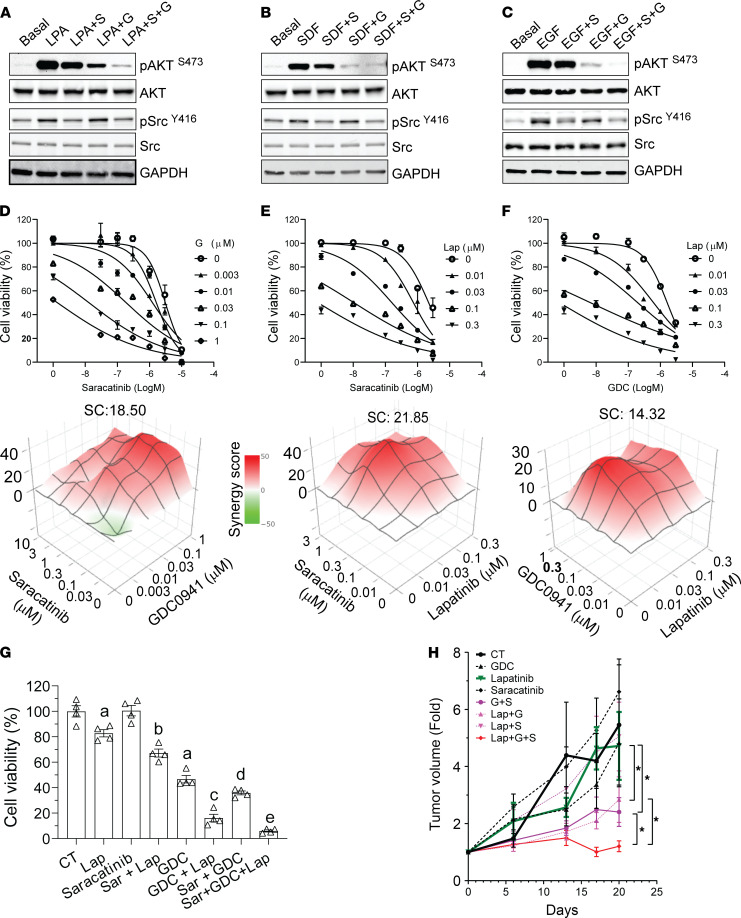
The combined effects of lapatinib plus PI3K and Src inhibitors on HER2^+^ breast cancer cells and tumors. (**A**–**C**) Effects of the PI3K and Src inhibitors, saracatinib (S) and GDC0941 (G), either alone or in combination, on LPA- (**A**), SDF1α- (**B**) and EGF-stimulated (**C**) AKT and Src signaling in Neu cells. The blot images were assembled from multiple blots run with samples from the same experiments. (**D**–**F**) Effects of different drug combinations, saracatinib and GDC0941 (**D**), saracatinib and lapatinib (Lab) (**E**), and GDC and lapatinib (**F**), on Neu cell growth. The dose-dependent inhibition curves and 3D plots for different drug combinations at varying concentrations are shown on the top and bottom, respectively. The synergy score (SC) for each drug combination is also shown. (**G** and **H**) Effects of lapatinib, saracatinib, GDC0941, and their combination on Neu cell (**G**) and tumor (**H**) growth. In **G**, a, b, c, d, and e indicate a statistically significant difference (*P* < 0.05, *n* = 4); a represents difference from control (CT), b represents difference from lapatinib, c represents difference from lapatinib alone and GDC alone, d represents difference from saracatinib alone and GDC alone, and e represents difference from saracatinib plus GDC (Sar+GDC). In **E**, 1-way ANOVA, *P < 0.05, *n* = 5–8.

The PI3K/AKT and Src signaling pathways are critical for HER2-induced breast cancer progression and resistance to HER2-targeted therapy (28–32). PI3K/AKT and Src are also the major pathways activated by many G_i/o_-GPCRs, so we asked whether targeting PI3K/AKT and Src signaling mimicked the inhibitory effect of blocking G_i/o_-GPCRs by PTx. To do this, PI3K/AKT and Src signaling was blocked using a pan-PI3K inhibitor, GDC0941, and a Src-selective inhibitor, saracatinib. As shown in Figure 6, A–C, these inhibitors selectively abolished EGF-, LPA-, and SDF1α-stimulated AKT and Src phosphorylation in Neu cells, although saracatinib caused a slight inhibition of EGF-, LPA-, and SDF1α-stimulated AKT phosphorylation, probably due to crosstalk between the Src and PI3K/AKT pathways.

When given alone, GDC0941 and saracatinib inhibited the proliferation of all breast cancer cells tested in a dose-dependent manner (Figure 6, D–F). Notably, overexpression of a constitutively active mutant of either AKT2 (myristoylated AKT2) or Src (GFP-tagged Src/Y527F) in Neu cells did not significantly enhance cell proliferation but completely abolished PTx-inhibited cell growth (Supplemental Figure 6, A and B), suggesting that G_i/o_-GPCR signaling promotes breast cancer cell growth, at least in part, through the redundant function of the AKT and Src pathways.

Synergism among GDC0942, saracatinib, and trastuzumab, or lapatinib was assessed, under varying concentrations, by checkerboard assays followed by analysis using the Bliss independent method. In Neu, MCF10A/HER, BT474, and BT474R cells, combining GDC0941 with saracatinib gave a synergistic effect (synergistic score >10; Figure 6D and Table 3). The combination of either GDC0941 or saracatinib with trastuzumab or lapatinib was also synergistic (Figure 6, E and F, and Table 3). A triple combination of lapatinib, saracatinib, and GDC0941 inhibited Neu cell growth more than combining any two (Figure 6G). Together, these findings suggest the PI3K and Src inhibitors enhance the therapeutic efficacy of trastuzumab and lapatinib.

The in vivo efficacy of combining the PI3K and Src inhibitors with lapatinib was tested against Neu syngeneic tumors. FVB mice were implanted with Neu cells that grew into tumors approximately 100 mm^3^ in size, after which mice were treated by daily oral gavage with vehicle control, lapatinib (150 mg/kg), GDC0941 (50 mg/kg), or saracatinib (10 mg/kg) alone or in combination, i.e., GDC0941 (50 mg/kg) and saracatinib (10 mg/kg), lapatinib (150 mg/kg) and GDC0941 (50 mg/kg), lapatinib (150 mg/kg) and saracatinib (10 mg/kg), or lapatinib (150 mg/kg), GDC0941 (50 mg/kg), and saracatinib (10 mg/kg), for 3 weeks. Under these conditions, lapatinib, GDC0941 or saracatinib alone, or lapatinib plus saracatinib had no significant effect (Figure 6H), whereas a combination of GDC0941 with saracatinib or lapatinib partially suppressed tumor growth. Strikingly, however, the combination of all 3 drugs not only blocked tumor growth but also caused tumor regression (Figure 6H).

## Discussion

Although multiple GPCRs are implicated in driving breast cancer formation and progression, the mechanisms underlying GPCR alteration in cancer are largely unknown (33). Moreover, to date, no approaches effectively targeted multiple GPCRs as a cancer therapeutic (7, 11). Our studies show why this has been so difficult: in breast cancer cells, expression of a multiplicity of GPCRs is altered, a finding consistent with reports that various tumor types differentially express more than 50 GPCRs (8). Moreover, our data show HER2 overexpression can alter GPCR expression in both mouse and human mammary epithelial cells, suggesting HER2 signaling is a key regulator of GPCR mRNA expression. However, given that only a small number of G_i/o_-coupled GPCRs (9) are similarly upregulated in Neu and MCF10A/HER2 cells, other signaling pathways likely also regulate GPCR expression in a cell-specific manner. The upregulated GPCR expression would contribute to aberrant GPCR signaling in tumors, because stimulation of the upregulated receptors enhances AKT and Src signaling (as with LPAR and CXCR4 and CXCR7 in MCF10A/HER cells). Because constitutively active mutants of GPCRs are relatively rare in breast cancer (8), our results suggest that GPCR upregulation by oncogenic signals may be the key mechanism contributing to aberrant GPCR signaling in tumors.

Our results are consistent with those of previous studies (8) and argue that G_i/o_-coupled receptors are the main group of GPCRs with altered expression in breast cancer cells. Many members of the G_i/o_-GPCR family, such as LPAR, CXCR4, CXCR7, and PAR1, play a role in breast cancer progression. In addition, the integrin-associated protein, CD47, can also function through the G_i/o_ proteins to regulate breast cancer cell growth (34). Nevertheless, targeting any one receptor as a cancer therapy has not achieved optimal efficacy in many clinical trials, likely because GPCRs function redundantly in promoting tumor progression. Our studies indicate that although not all G_i/o_-GPCRs are upregulated in tumor cells, the overall function of G_i/o_-GPCRs is to promote breast cancer formation and progression. This is evident, in that uncoupling G_i/o_-GPCRs from G_i/o_ proteins by PTx suppresses tumor cell growth and migration in vitro and blocks tumor formation and progression in vivo. PTx effects are likely specific, as it selectively inhibits G_i/o_-GPCR–mediated but not EGF-stimulated signaling and does not affect normal mammary gland development. Nevertheless, because G_i/o_ proteins also mediate the function of other cell surface receptors (e.g., CD47), we cannot exclude the possibility that some of the effects of PTx are mediated by other receptors. Our findings indicate that G_i/o_ signaling contributes to breast cancer growth and metastasis and might be a good target for therapeutically blocking breast tumor progression. As a demonstration of the potential power of this approach, we showed that blocking G_i/o_ signaling by PTx enhances the therapeutic efficacy of HER2-targeted therapy both in vitro and in vivo. Notably, blocking G_i/o_ signaling also sensitizes trastuzumab-resistant cells to trastuzumab, suggesting that targeting G_i/o_ signaling may represent a new approach to overcome resistance to HER2-targeted therapy.

Although PTx is a valuable tool for dissecting the function of G_i/o_-coupled receptors, it cannot be used as a therapeutic agent for blocking G_i/o_ signaling because it is a virulence factor of Bordetella pertussis and causes the respiratory disease pertussis (14). G_i/o_-GPCRs transmit signals through Gα_i/o_ and Gβγ subunits; signals originating from both are implicated in tumor progression, suggesting that directly targeting G proteins may be an approach to block G_i/o_-GPCR signaling (6, 13). Yet, no Gα_i/o_-specific inhibitors are currently available (7). Although several small inhibitors of Gβγ have been developed, they may not selectively target G_i/o_-GPCR signaling because other classes of GPCRs can also initiate Gβγ signaling (35). Moreover, although Gβγ signaling is critical for growth and metastasis of triple-negative breast cancer cell lines (17, 22), whether it mediates progression of HER2-driven breast cancer remains unknown.

An alternative approach to targeting G_i/o_-coupled receptors is to target their shared signaling pathways that are essential for cancer development and progression. Our results suggest that PI3K/AKT and Src signaling represent such pathways and so might be therapeutically targeted in HER2^+^ breast cancer. First, as reported here and previously (36–39), PI3K/AKT and Src can be activated downstream of multiple G_i/o_-GPCRs. The G_i/o_-GPCR–mediated mechanisms for PI3K/AKT and Src activation are complex and likely receptor dependent. PI3K/AKT and Src may be activated by direct interaction with Gα_i/o_ and Gβγ subunits or may be downstream of other signaling molecules such as small G proteins (36–40). Our data suggest that PI3K/AKT and Src signaling have a redundant function in mediating G_i/o_-GPCR–regulated tumor cell growth, because expression of either an active AKT or Src mutant is sufficient to block the inhibitory effect of PTx on cell growth.

Second, PI3K/AKT and Src are key pathways activated by EGFR and HER2. PI3K/AKT and Src activities play key roles in HER2-induced breast tumor formation and progression (28–32). Moreover, they appear to be the convergence points for crosstalk between G_i/o_-GPCRs and EGFR/HER2 (41–43). Although G_i/o_-GPCRs induce transactivation of EGFR and HER2, the activation of EGFR and HER2 in turn contributes to G_i/o_-GPCR–mediated signaling. Although the underlying mechanisms are unclear, the contribution of EGFR and HER2 transactivation to GPCR signaling appears to be pathway specific, because blocking EGFR significantly reduced LPA- and SDF1α-stimulated AKT phosphorylation but had little effect on Src phosphorylation. Finally, several studies suggest that aberrant PI3K/AKT and Src activation contributes to resistance of HER2^+^ breast cancer to HER2-targeted therapy (32, 44–47). Consistent with these results, in breast cancer cell lines overexpressing HER2, the combination of PI3K or Src inhibitors with HER2-targeted therapy synergistically inhibits growth (44–47). Notably, recent reports demonstrate that resistance to combined inhibition of PI3K and HER2 in HER2^+^ breast cancer is associated with upregulation of collagen, integrin β1, and Src activity (48), suggesting that inhibiting both PI3K and Src may overcome resistance to the HER2-targeted therapy better than inhibiting just PI3K or Src alone. Our data support these findings and provide the first evidence to our knowledge that combined inhibition of both PI3K and Src synergizes to block tumor growth and sensitize tumors to HER2-targeted therapy.

In conclusion, our study provides several potentially novel findings. It showed for the first time to our knowledge that HER2 overexpression alters expression of multiple GPCRs in breast cancer cells and demonstrated that G_i/o_-GPCRs, the largest subset of GPCRs, promote HER2-induced breast cancer formation and progression in a genetic mouse model of HER2^+^ breast cancer. Moreover, we provided a proof of concept that the G_i/o_-GPCRs can be targeted, as a whole, to overcome the redundant function of multiple GPCRs and to block tumor progression and enhance HER2-targeted therapy. Finally, our evidence shows that PI3K and Src signaling represent the pathways shared downstream of G_i/o_-GPCRs that may be targeted to enhance the efficacy of the HER2-targeted therapy. Because a common feature of all breast cancer subtypes is overexpression of multiple GPCRs, our findings have important implications for how to target GPCRs therapeutically in other breast cancer subtypes.

## Methods

### Reagents.

LPA and PTx were from MilliporeSigma. Human and mouse SDF1-α were from Pepro Tech. EGF was from Gold Biotechnology. Sapitinib, erlotinib, and cp-724714 were from Shelleck Chemicals. Trastuzumab was from Genetech. Lapatinib, GDC0941, and saracatinib were from LC Laboratories. Antibodies for EGFR (no. 2232), phospho-EGFR^Y1068^ (no. 3777), HER2 (no. 2165), phospho-HER2^Y1221/1222^ (no. 2243), AKT (no. 4685), phospho-AKT^S473^ (no. 4060), Src (no. 2109), phospho-Src^Y416^ (no. 6943), ERK1/2 (no. 4696) and phospho-ERK1/2^T202/Y204^ (no. 4370) were from Cell Signaling Technology. GAPDH (sc-47724) was from Santa Cruz Biotechnology. Ki67 (GTX16667) was from GeneTex. EGFR (no. 4267) from Cell Signaling Technology and phospho-Src^Y418^ (ab4816) from Abcam were used in immunohistochemical staining. Antibodies for LPAR1 (NBP1-03363SS), CXCR4 (NB100- 56437SS), and CXCR7 (NBP2-24779SS) were from Novus Biologicals.

### TCGA data analyses.

The cBioportal for Cancer Genomics (https://www.cbioportal.org/) was used to analyze the mRNA expression of approximately 400 nonsensory GPCRs in the breast invasive carcinoma data set (TCGA, PanCancer Atlas). A 2-fold *Z*-score threshold identified patients with altered GPCR mRNA expression levels, as compared with diploid samples. The results are presented in heatmaps according to the molecular subtypes of breast cancer and G protein linkage of GPCRs (Supplemental Figure 1). The linkage of GPCRs to G proteins was assigned based on information for GPCRs in the IUPHAR/British Pharmacological Society website.

### Cell lines.

BT474 and MCF10A cells were purchased from the ATCC. The trastuzumab-resistant BT474 derivative (BT474R) and HER2-overexpressing MCF10A (MCF10A/HER2) cells were provided by Hank Qi (Department of Anatomy and Cell Biology, University of Iowa). Neu cells were generated from tumors arisen from the transgenic mice, MMTV-c-Neu mice, and cultured in DMEM media containing 10% FCS. Cell lines were tested for *Mycoplasma* using the Mycoplasma Detection kit (ATCC). BT474 and BT474R cells were cultured in DMEM/F12 media containing 10% FCS. MCF10A and MCF10A/HER2 cells were cultured in DMEM/F12 media containing 5% horse serum supplemented with EGF at 20 ng/ml, hydrocortisone at 500 ng/ml, cholera toxin at 100 ng/ml, and insulin at 10 μg/ml. Each cell line was cryopreserved at low passage numbers (fewer than 6 passages after receipt) and used in experiments for a maximum of 18 passages.

### Isolation of mammary epithelial cells.

Mammary epithelial cells were isolated from the mammary glands of 4-month-old wild-type and MMTV-c-Neu transgenic FVB/N mice, using the EasyStep Mouse Epithelial Cell Enrichment Kit (StemCell Technologies).

### Analysis of gene expression.

Total RNA was extracted from the isolated mammary epithelial cells pooled from 4 mice and MCF10A and MCF10A/HER2 cells using a Qiagen RNeasy MinitKit. cDNA was synthesized from 2 μg total RNA using a High-Capacity cDNA Reverse Transcription Kit (Applied Biosystems). The expression of 370 mouse nonsensory GPCRs was analyzed using the RT2 Profiler PCR array (Qiagen, 330231), as per the manufacturer’s instructions. 106 human G_i/o_-GPCRs and the effects of PTx were analyzed with real-time qPCR using the SensiFAST SYBR No-ROX kit (Medidian Bioscience) and specific primers designed and synthesized by Integrated DNA Technologies (Supplemental Table 1). C_t_ values of target genes were normalized to the mean C_t_ values of the housekeeping genes, actin, β2 microglobulin, glyceraldehyde-3-phosphate dehydrogenase, β glucuronidase, and heat shock protein 90 α. The expression of a gene was considered undetectable if its C_t_ value was less than 35. The fold changes of gene expression relative control are presented using the Graphpad Prism Software.

### Plasmids.

The lentiviral vector (Tet-CA-Src-GFP) for tetracycline-inducible expression of the GFP-tagged, constitutively active Src mutant, Src/Y527F, was obtained from Addgene (item no. 83469). The lentiviral vector for tetracycline-inducible expression of myristoylated constitutively active AKT2 mutant was constructed by first cloning the myristoylated HA-tagged AKT2 from pcDNA3 (Addgene, 9016) to pENTR vector (Thermo Fisher Scientific) and then to the destination vector pLIX403 (Addgene, 41395) by the Gateway cloning.

### Lentiviral production.

Lentiviruses were generated in HEK293FT cells as described previously (23). Lentiviruses collected from the culture supernatants were concentrated using the Lenti-X-concentrator (Takara Bio).

### Establishment of stable cell lines.

The Neu cells were transduced with lentiviruses encoding GFP-tagged Src/Y527F or myristoylated-HA-AKT2 and selected with puromycin (2 μg/ml) for at least 1 week to establish stable lines.

### Cell proliferation and viability assays.

Cell proliferation in 2D culture or in Matrigel was analyzed as we described previously (17, 23). To analyze the response of cells to drug treatment, 3000 cells were seeded in triplicate into a 96-well plate, and varying concentrations of drugs were added the next day. Cells were exposed to trastuzumab for 5 days or other inhibitors for 3 days. Cell viability was quantified using AlamarBlue assays (Thermo Fisher Scientific), as per the manufacturer’s instructions. For combinational studies of 2 drugs, percentage inhibition was calculated and analyzed using the SynergyFinder 2.0 software (49).

### Cell migration and wound-healing assays.

Transwell migration and wound-healing assays were performed as we described previously (17, 23). To exclude the influence of cell proliferation, cells were treated with 5 μg/ml mitomycin.

### Cell stimulation.

Cells were seeded into 6-well plates. After 48 hours of serum starvation, cells were pretreated with the indicated inhibitors for 1 hour and then stimulated with various agonists for the indicated time. To determine the effect of PTx, cells were treated with PTx (200 ng/ml) for 24 hours.

### Western blot analysis.

Protein lysates were prepared from cells and tumor tissues and analyzed by Western blotting as we described, using the iBright CL1000 (Thermo Fisher Scientific) or Odyssey (LI-COR Biotechnology) imaging system (17, 23).

### Mouse studies.

MMTV-c-Neu mice were from The Jackson Laboratory (no. 005038). TetO-PTx mice were from the Mutant Mouse Resource & Research Center (no. 014241), and MMTV-tTA mice were generated in-house laboratory (50). All mice were in the FVB/N genetic background. Mice were genotyped by PCR as reported previously (24, 50, 51). Female mice were kept as virgins throughout the experiments. To determine tumor onset, starting at 4 months after birth, mice were checked twice per week by palpation. To determine tumor progression, the largest tumor was measured weekly by caliper. To determine lung metastasis, the lung was harvested once the largest tumor reached a size of 2 cm in diameter and was perfused and fixed with 4% paraformaldehyde before paraffin embedding. The number of metastases in the lung was analyzed by serial sectioning followed by H&E staining.

To generate syngeneic tumor models, tumor cells were isolated from size-comparable tumors arisen from transgenic mice, i.e., MMTV-c-Neu/MMTV-tTA (Neu) and MMTV-c-Neu/MMTV-tTA/TetO-PTx (PTx), using the Mammary epithelial cell enrichment kit (StemCell Technologies). 1 × 10^6^ cells were injected into the right inguinal mammary fat pads of FVB/N mice. To determine the effect of PTx expression on tumor growth, mice were fed with normal chow or doxycycline-containing chow (625 mg/kg; ENVIGO) to turn off PTx expression. Two months after tumor cells were implanted, tumors were excised and weighed. To determine the response of tumors to drug treatment, tumors were grown to a size of approximately 100 mm^3^ and then treated with vehicle or drugs for the indicated time. Tumor growth was monitored twice per week by caliper measurement.

### Whole-mount, histology, and immunohistochemical analyses.

Whole-mount staining of mammary glands from mice of different ages and histological and immunohistochemical analyses of tumors were performed as described (23, 52). Ki-67 was quantified by counting the number of positive nuclei for every 100 nuclei from multiple fields on the slide to obtain the percentage of positive cells.

### Statistics.

Data are expressed as mean ± SEM. Statistical comparisons between groups were analyzed by 2-tailed Student’s *t* test or 1-way or 2-way ANOVA, as indicated in the figure legends. *P <* 0.05 was considered significant.

### Study approval.

All animal studies were conducted in accordance with an IACUC-approved protocol at the University of Iowa.

## Author contributions

CL and ML curated the data. CL, ML, and SC provided formal analysis. KW provided critical materials. CL and ML provided methodology. RW and SC provided project administration. SC wrote the original draft. RW and SC reviewed and edited the manuscript.

## Supplementary Material

Supplemental data

Supplemental table 1

## Figures and Tables

**Table 1:**
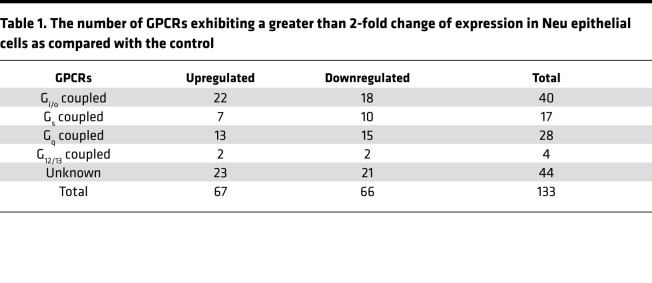
The number of GPCRs exhibiting a greater than 2-fold change of expression in Neu epithelial cells as compared with the control

**Table 2:**
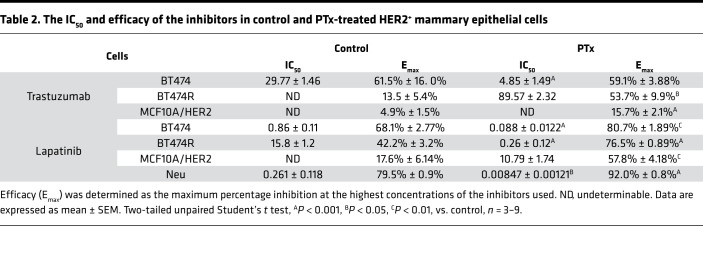
The IC_50_ and efficacy of the inhibitors in control and PTx-treated HER2^+^ mammary epithelial cells

**Table 3:**
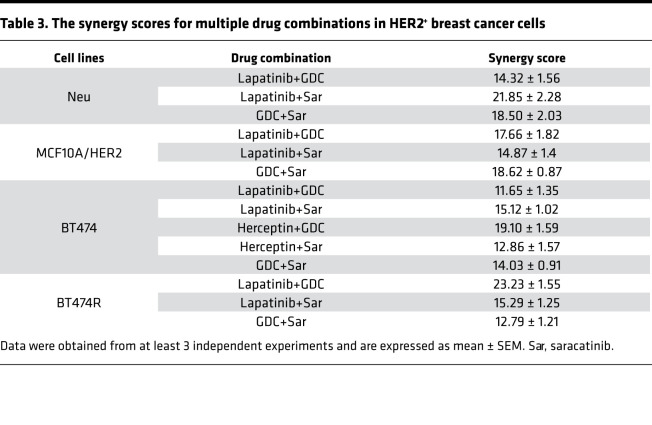
The synergy scores for multiple drug combinations in HER2^+^ breast cancer cells

## Version 1 08/03/2021

In-Press Preview

## Version 2 09/22/2021

Electronic publication
