# Supplementary material for: Targeting Gi/o protein–coupled receptor signaling blocks HER2-induced breast cancer development and enhances HER2-targeted therapy
Source: JCI Insight. 2021 Sep 22;6(18):e150532. doi: 10.1172/jci.insight.150532 (PMC8492335; doi:10.1172/jci.insight.150532)
Supplement: Supplemental table 1 [file jciinsight-6-150532-s240.pdf]

Supplemental Table: The sequences of the primers used for real-time qPCR analysis of human $G_{i/o}$-GPCRs. F, forward; R, reverse.

Gene Name	Sequence
ADORA1-F	GGC ATG TGA GGA CTG GAC
ADORA1-R	GAA TGA CCA TTT CCG CTG C
ADORA3-F	CAT ACA GGC CCT CAA GTG TT
ADORA3-R	TGC CAT CCC TCT GAT TCT TTG
ADRA2B-F	ACA CAG CAG GGC AAG AAG
ADRA2B-R	CTA CAC CAT CTT CAA CCA GGA C
ADRA2C-F	CGG GTC TCT GGG AAA GC
ADRA2C-R	TTA AGC ACA TCC TCT TCC GAC
AGTR2-F	AGC AAG CTG ATT TAT GAT AAC TGC
AGTR2-R	ACA AGC CCG AAG TGA AGA C
APLNR-F	AAG GGT GGA GAA CAG ATG C
APLNR-R	TTT CAG AAA GCA AGG GCA AAG
C3AR1-F	GAG ACA TCC AGG TGC TGA AG
C3AR1-R	GCT GAG AAT GAC CAT GGA GAG
C5AR1-F	GGT TAG GGA GAG CAA GTC ATT C
C5AR1-R	GAA AAC AAG AGG GAG AAT GGC
CASR-F	AGC ACC TAC GGC ATC TAA AC
CASR-R	ATA CCC GAC TTC CTT AAA CAC G
CCR1-F	CAG AAA GCC CCA GAA ACA AAG
CCR1-R	TGC ATC CCC ATA GTC AAA CTC
CCR2-F	GAT CTG TTT GGT TCA GTT GCT G
CCR2-R	AAA CCG AGA ACG AGA TGT GG
CCR3-F	GCT ATC ACT GGA CAT ATC AAG GAC
CCR3-R	CTC TGT TAC CAG CTT CTC ATC TG
CCR4-F	CTT CCT GAG CAA GCC TGG
CCR4-R	GTG TCT GCT ATA TCC GTG GG
CCR5-F	CCT ACA AGA AAC TCT CCC CG
CCR5-R	ACC AGT GAG TAG AGC GGA G
CCR6-F	CTG TGG CTG TTG GTT TGT G
CCR6-R	AAA GGA GTG TAT GGT TCA GCC
CCR7-F	GGG AAA CCA ATG AAA AGC GTG
CCR7-R	GGA GCA CAA AGA CTC GAA C
CCR8-F	GGA ATT GGC AAC ACT GAA ACC
CCR8-R	ATA AGT TCC GCA TCA CAG GG
CCR9-F	CAG ACA CTG AGA GCT GGT G
CCR9-R	TGG AAG ATG TGG ATT CAG AGC
CCR10-F	CCT TTC TTC CTG CTC AGC TC
CCR10-R	TGC CTC TTT CTC AGT GTT CC
CCRL1-F	CTC TGT ACT CAA GAC TGC TCC
CCRL1-R	CTG TTG ACT GGT TCT GTT CC
CCRL2-F	ATA CCT CTG CCG CTG TTT C
CCRL2-R	TTC TTC TTG CAT TTG GTG GAT G

CHRM2-F	GTT AGC GAC ATG AGT ACA GGG
CHRM2-R	TTG CGT TCT CTA ATC AGT AGC C
CHRM4-F	TCA ACA GCA CCA TCA ACC C
CHRM4-R	GGA ACT CCT GCC TGC CTA
CNR1-F	GGG TCA CTT TCT CAG TCA TTT TG
CNR1-R	TCA TTT GAG CCC ACG TAC AG
CNR2-F	AGA AGC CCC GAG ATC CTC
CNR2-R	CCA GGG AGT GAA CTG ATT TCT G
CX3CR1-F	AGG CAT GGA AGT GTT CTG AG
CX3CR1-R	CTA GTC AGC ATC AGG TTC AGG
CXCR1-F	GCC CTC TAG CTG TTA AGT CAC
CXCR1-R	GTC CTC TTC AGT TTC AGC AAT G
CXCR2-F	GTC TAA CAG CTC TGA CTA CCA C
CXCR2-R	TTA AAT CCT GAC TGG GTC GC
CXCR3-F	CAA CCA CAA GCA CCA AAG C
CXCR3-R	GGA AGA GCT GAA GTT CTC CAG
CXCR4-F	AGC AGG TAG CAA AGT GAC G
CXCR4-R	CCT CGG TGT AGT TAT CTG AAG TG
CXCR5-F	GGA GCC TCT CAA CAT AAG ACA G
CXCR5-R	GGG AGG TGT CGT TAT AGT TGT C
CXCR6-F	GGA ACA AAC TGG CAA AGC ATC
CXCR6-R	GAA AGA CCT TGC TGA ACT GC
CXCR7-F	GCC TTC ATC TTC AAG TAC TCG
CXCR7-R	CAA GTA AAC CCG TCC CAG AG
DARC-F	CTT CTC ATC TGG ACA CCC TTG
DARC-R	GCA GTG TAG ACT TTA ATT CAG GTT G
DRD2-F	GTC ATG ATC TCC ATC GTC TGG
DRD2-R	AAT GAA GGG CAC GTA GAA GG
DRD3-F	TCG ACA TCT TTG AAG CTG GG
DRD3-R	AGT GGG TAT TGA GAA CAT GGG
DRD4-F	CAA CCC CGT CAT CTA CAC TG
DRD4-R	CAT CTC CTT GGT CCC TGA G
EDNRB-F	CAA GGA CCC ATC GAG ATC AAG
EDNRB-R	AGA TAT TGG GAC CGT TTC GC
F2RL1-F	TCA CCT CAA AGA AAC ACT CCA G
F2RL1-R	GAC ACG TCC TCA TAA CAT TAA ACA G
F2RL3-F	CCT CTT TGC TCC AGT GAC AC
F2RL3-R	CAA AGT GAC CTC CGC TAG TG
FFAR2-F	TGT ATG GAG TGA TTG CAG CTC
FFAR2-R	TGG TTA TCG GTG AAG TTC TCG
FFAR3-F	CCT GTG CTG AAA GCT AGG TC
FFAR3-R	TTA GGG AGG CTA GAT GTC TAC TC
FPR1-F	AGA CCT AGA ACT ACC CAG AGC
FPR1-R	GAG ATA GCC AGC AGA TAC AGC
FPR2-F	CAA TAT GGA TTT GCA CCC ACT G

FPR2-R	AGG AGT GGA GAA GTT GGT TTC
FPR3-F	TGG GCA CAG GAA AAG GAT C
FPR3-R	CCG TGG ACT AGC AAT GAG AAG
GALR1-F	ACA TTC GCA AAG ATT CAC ACC
GALR1-R	TGT TTC TGT GTC TGG TCC AC
GALR2-F	CAG CCA TGC ATC CTC GAG
GALR2-R	GAC TCC AAC TCT GTG ACA TCC
GRM2-F	ACT GGT GTT ATT GGC GGT TC
GRM2-R	CAT AGC GGG ACT TGT CAC TC
GRM3-F	ACC TCT TTT GTG TCG GAT GAG
GRM3-R	GAG GAA CAG ACT AAC AGA GCC
GRM6-F	TCA CCA TCC TGC CCA AAA G
GRM6-R	AAA TTC TCT TCC CAG AAC TCG G
GRM7-F	GGA ATT ATG TGT CTA CCC TCG C
GRM7-R	TCC TGT CTT TGC GTT CCT G
GRM8-F	GAA CTA TGG TGA GAG CGG TG
GRM8-R	ATT AGG TGT TTC TAG CAG GCG
HCAR1-F	GTT TCC AAA GCC AGT CTG ATG
HCAR1-R	ACC CCT TAG CAC GAG TTA ATT C
HCAR2-F	CTC ACT AGC CGC ACT CAT G
HCAR2-R	AAG CCC GAA GAT AAA CTC CAG
HRH3-F	CTC AAC CTC GCC ATC TCC
HRH3-R	GTC CAC TAC CAG CCA CAG
HRH4-F	TGC TAG GAA ATG CTT TGG TCA
HRH4-R	CAG CGT GTG AGG GAT GTA C
HTR1A-F	GAG AGG AAG ACA GTG AAG ACG
HTR1A-R	AGA ACA AGA GCC ACG ATG AAG
HTR1B-F	ATC CTC TAC ACG GTC TAC TCC
HTR1B-R	GCG TCT GTT TCA AAA TCC GG
HTR1D-F	AAA TGT GTG GAG GTC TGT GG
HTR1D-R	TCT GAG GTT TCT GTG GCA TTC
HTR1E-F	AAA GCT CAT TAG ATG CCG AGA G
HTR1E-R	ACA TAC TTA CAA ACT CTC CTG AAC C
HTR1F-F	CTG GAG GGC GTT TCA AGA G
HTR1F-R	AGT TTT GAT CAG ATG AAT TTA AGA AAT CC
HTR5A-F	CCC CTG ATC TAT ACG GCT TTC
HTR5A-R	TGG GAA ATG GAT GAA CTG GG
LHCGR-F	GAA GCT AAT GCC TTT GAC AAC C
LHCGR-R	GGA AAC TTT CTG ATG CCT GTG
LPAR1-F	AAT TTG TCT CCC GTA GTT CTG G
LPAR1-R	ATG GAC TCG TTG TAG AAG CAC
LPAR2-F	AGC CTG GTC AAG ACT GTT G
LPAR2-R	CGG CCA ACA GTA GGA AGT AC
LPAR3-F	AGG ACA CCC ATG AAG CTA ATG
LPAR3-R	GGA ACC ACC TTT TCA CAT GC

LPAR4-F	GCG AGT TGC CCA TTT ACA TG
LPAR4-R	CAC TCT GAC ACT ATG GAC CG
LTB4R-F	TTT GGC AGT GGG ACA GAT C
LTB4R-R	AGA TGT AGT GTT CAT GGC CG
LTB4R2-F	AGG AGT TGT GTT TGA GGT GG
LTB4R2-R	CTG TGA TGC CCG ATG AGA AG
MCHR1-F	AGC AAC ACC TCT GAT GGC
MCHR1-F	GAA GAT GAC CGT GGA GTT CC
MRGPRD-F	CAT CTA TAT CCT CAA CCT GGC G
MRGPRD-F	AGG CAA AGT ACA TCA GTC TCT TC
MTNR1A-F	GTC ATC CTG TCG GTG TAT CG
MTNR1A-R	AAC GGG TAC GGA TAA ATG GC
MTNR1B-F	CAT TCA AGA TGC TTC CAA GGG
MTNR1B-R	ACT CTC TCC CAC CAT TTC ATG
NPBWR1-F	AAC CTG TTC ATC CTC AAC CTG
NPBWR1-R	GTT GTA CTG GTC GAT AGC CAC
NPBWR2-F	CGT TCC TCT ATG TGC TCC TG
NPBWR2-R	GTC ACC GTC TTC ATC TTG GG
NPFFR2-F	TCG CAG CTT CAG TCT TTA CG
NPFFR2-R	TGG AGA CAT AAT GGT GAT GGC
NPR1-F	ACA GTC AAC ACA GCC TCA AG
NPR1-R	CCT TCA TTT CTA CAT CCC CTC G
NPR2-F	AGA TCT TTG CCA ACA CCG G
NPR2-R	TGA AGC GAG TGA GAT GGT TG
NPR3-F	TCT CCC TGC AAA TCA TGT GG
NPR3-R	TCG CCT CTC AAT GGT TAT TCT G
NPY1R-F	CAA GCC CAG TCG CAT TTA AAA
NPY1R-R	CAG GTA ATC AAA GTA TGT TGC AGG
NPY2R-F	ACA TCT TGT TTC CGC GTC TC
NPY2R-R	CCC ATT TTC AGT ACA GGT CCA C
NPY5R-F	AAG GGA AAG GGT GTT ACA AGG
NPY5R-R	ATT CTC TGT GGC AAG TGT CTT
OPRD1-F	TGT CAT GTT CGG CAT CGT C
OPRD1-R	CAT CAG GTA CTT GGC ACT CTG
OPRK1-F	TCG CTG GTC ATG TTC GTG
OPRK1-R	CCA AAA GGC CAG GAA TTC ATC
OPRL1-F	CAC AGG TCG AGG ATG AAG AG
OPRL1-R	GAT CAT GAG GCT GTA GCA GAC
OPRM1-F	TCT CTT CAG CCA TTG GTC TTC
OPRM1-R	GAA GGC GAA GAT GAA AAC ACA G
OXTR-F	CCT TTG TCC TGA GCC ATC G
OXTR-R	CAC AAA CAT ACG CCA TCA CC
P2RY12-F	ATA AGT ATG CTG AAG TGC CCC
P2RY12-R	CGA GTT CTG AAC ACA AAG AGA TTG
P2RY13-F	TGT AGG CTG AAC TAA TGA CTG C

P2RY13-R	TGT ACT ATC CGA GTG TCT CTG G
PTAFR-F	TGA TCA TTC CAG CCC ACA G
PTAFR-R	AAA GAC CCA CAG CAC GTA G
PTGER3-F	AGA CGG CCA TTC AGC TTA TG
PTGER3-R	TCT TTC TGC TTC TCC GTG TG
RXFP1-F	AGT GCT CCC TTG GCT ATT TC
RXFP1-R	GTC AAA TTG CAG AGA CCA TCC
RXFP2-F	ACA ACT CAC GCT ATG TCC ATC
RXFP2-R	CAT ACT TCT GAT ACT GCC CTC G
RXFP3-F	CGT GTT ATC TTA GGT CTT GTC CC
RXFP3-R	AAG AGT TCT GCT AGC TTG TCC
RXFP4-F	AAT CTC TGA TGC CCT GCG
RXFP4-R	AGG AAT TTG ACA GGC ATC GG
S1PR1-F	TCG AGT AAG TTT GCG AGA GC
S1PR1-R	CTT TTC CTT GGC TGG AGA GG
S1PR3-F	AGC ACT TCA GAA TGG GAT CTT C
S1PR3-R	GGT CAA AGT AAG GTA GCT CTC C
S1PR4-F	GCT TCC ACC ACC GAC AG
S1PR4-R	ACC ATC CAC ACG CAA GAC
S1PR5-F	GGT ATC AGA ACC GGC TGC
S1PR5-R	CGC TGC ATA AAT ACA TTT CCC C
SSTR1-F	TCT TCC GTA ATG GCA CCT G
SSTR1-R	GAT ACC GTC CCT TTC GCA C
SSTR2-F	GGA GCG GAG TGA CAG TAA G
SSTR2-R	CCC ATT GCC AGT AGA CAG AG
SSTR3-F	GCG CAT CAG CTA CCT GTA G
SSTR3-R	CAA TAG CAT CAA AGT CCA GGC
SSTR4-F	GTC GCT ATC CAG TGC ATC TAC
SSTR4-R	AGG TTG AGC AGG TAG ATG TTG
SSTR5-F	AAC ACG CTG GTC ATC TAC G
SSTR5-R	ACT GCC AGG TTG AGA ATG TAG
TSHR-F	AGT TTC CTT CAC CTC ACA CG
TSHR-R	ACT GCT CTC ATT ACA CAT CAA GG
XCR1-F	ACC ATT TCT GCC TCA AGA CG
XCR1-R	TGC CTA TAG TCC CAG CTA CTC
